# Towards a Rational Design of Biosensors: Engineering Covalently Grafted Interfacial Adlayers as a Testbed Platform for Electrochemical Detection of Epinephrine

**DOI:** 10.3390/molecules30102236

**Published:** 2025-05-21

**Authors:** Xiaoli Chang, Yuan Fang, Oleksandr Ivasenko

**Affiliations:** State Key Laboratory of Bioinspired Interfacial Materials Science, Institute of Functional Nano & Soft Materials (FUNSOM), Soochow University, Suzhou 215123, China

**Keywords:** electrochemical biosensor, diazonium grafting, catecholamines, graphene functionalization, electrochemical interface design

## Abstract

The performance of electrochemical (bio)sensors is fundamentally determined by the precise engineering of interfacial layers that govern (bio)analyte–surface interactions. However, elucidating structure–function relationships remains challenging due to the complex architecture of modern sensors and the irregular nanoscale morphology of many high-performance materials. In this study, we present a strategy for designing custom functional interfaces as well-defined platforms for probing interfacial processes. Focusing on epinephrine (EP) detection as an important representative of catecholamines, we compare the interfacial behavior of two carboxy-functionalized electrodes—grafted with either para-aminobenzoic acid (PAB) or 3,4,5-tricarboxybenzenediazonium (ATA)—against atomically flat highly oriented pyrolytic graphite (HOPG) as a control. While both modifiers introduce carboxyl groups, PAB forms disordered multilayers that inhibit surface responsiveness, whereas ATA yields an ultrathin monolayer with accessible COOH groups. Electrochemical analysis reveals that ATA-HOPG significantly enhances EP detection at sub-micromolar levels, facilitated by electrostatic interactions between surface-bound COO^−^ and protonated EP and its redox products. These results demonstrate that nanoscale control of diazonium grafting is crucial for optimizing bioanalyte recognition. More broadly, this work highlights how molecular-level surface engineering on high-quality carbon substrates can serve as a test-bed platform for the rational design of advanced electrochemical sensing interfaces.

## 1. Introduction

Biosensors play a pivotal role in modern analytical science by providing powerful tools for the sensitive and selective detection of biologically relevant molecules [1,2,3]. By integrating biological recognition elements with signal transducers, these devices enable the rapid, real-time, and often label-free detection of a wide range of targets, from small metabolites and neurotransmitters to nucleic acids and proteins [4,5,6]. These capabilities prove essential in diverse fields such as medical diagnostics [7], environmental monitoring [8,9], food safety [10,11], and pharmaceutical development [12,13]. With the increasing demand for early disease detection, personalized medicine, and continuous health monitoring, the need for biosensing platforms with enhanced sensitivity, stability, and molecular specificity became increasingly urgent—particularly for detecting trace biomolecules in complex biological matrices [14,15,16].

Among the various materials employed in the construction of biosensors, carbon-based nanomaterials attract considerable attention due to their outstanding physicochemical properties. Materials such as graphene [17], graphene oxide (GO) [18], carbon quantum dots (CQDs) [19], and carbon nanotubes (CNTs) [20] exhibit excellent electrical conductivity [21,22,23], high mechanical strength [24,25,26], large specific surface areas [27,28], and remarkable chemical stability [29]. These features facilitate efficient electron transfer [30,31], high biomolecule loading capacity [32,33,34], and amplified signal responses [35,36], rendering carbon nanomaterials as ideal candidates for electrochemical biosensors [37,38,39]. Indeed, they are successfully applied to the detection of a variety of bioanalytes—including glucose [40,41], dopamine [42,43,44], epinephrine [45,46], and DNA [47]—demonstrating superior performance over conventional sensing materials.

Despite these advantages, a critical limitation of carbon nanomaterials lies in their lack of inherent molecular recognition capability. To impart target selectivity, surface functionalization is commonly employed by introducing specific chemical groups, receptors, or biomolecules that could selectively interact with the analyte of interest [44,46,48]. However, several challenges remain in developing functionalized carbon surfaces suitable for biosensing applications. First, precise control over the functionalization process at the molecular level proves difficult, often resulting in heterogeneous and poorly defined surface structures [49,50]. Second, the stability and reproducibility of the functionalized interfaces are often suboptimal, particularly under physiological or electrochemical conditions [51,52]. Third, the structure–performance relationship at the molecular interface remains poorly understood, hindering the rational design of efficient and robust sensing platforms [44]. These challenges highlight the urgent need for controllable, stable, and well-characterized functionalization strategies to fully exploit the potential of carbon nanomaterials in biosensor applications.

In response to these limitations, researchers have explored a variety of strategies to functionalize carbon nanomaterials, including non-covalent adsorption of polymers or biomolecules [53], and in situ growth of nanostructures such as metal–organic frameworks (MOFs) [54]. While non-covalent approaches preserve the intrinsic conductivity of carbon substrates, they often suffer from limited stability due to weak interactions with the surface. In contrast, covalent methods provide robust and permanent modification, but may partially disrupt the π-conjugation network of carbon materials, leading to reduced conductivity. To balance these trade-offs, recent studies have turned to electrochemical grafting using diazonium chemistry, which offers a controllable route to install diverse functional groups with tunable surface coverage and strong adhesion [55]. There are some successful applications of molecular design of grafted adlayers in the design of new sensors [56]. Also, we have developed a custom grafting formulation for the functionalization of chemically delicate graphene on ultra-thin layers of copper [57,58], which later were successfully used for the fabrication of ultra-sensitive plasmonic sensors of biotoxins [59] and malaria-specific DNA targets [60]. However, further investigation is required to better understand how the structural features of the grafted layers influence biosensor performance, especially in terms of recognition efficiency, electron transfer, and fouling resistance.

In this study, we applied a well-established covalent functionalization strategy based on the electrochemical grafting of para-substituted aryl diazonium salts onto highly oriented pyrolytic graphite (HOPG) to address the challenges of constructing stable and well-defined sensing interfaces. This strategy involves the electrochemical reduction of diazonium salts at the electrode surface to generate highly reactive aryl radicals, which rapidly form covalent bonds with the carbon substrate. The resulting modification is robust and versatile, enabling the incorporation of a wide variety of functional groups (e.g., –COOH and –NO_2_) through careful selection of diazonium precursors. While the grafting of aryl diazonium salts onto carbon materials has been extensively explored [55,61,62], we demonstrate the use of covalently grafted, ultrathin molecular adlayers as model interfaces to probe and understand electrochemical recognition processes relevant to biosensing. We started with para-aminobenzoic acid (PAB), which bears a single carboxylic acid group in the para position and has been widely used in previous studies, either in its native form to enhance the performance of composite-modified electrodes [46], or as a versatile linker for subsequent covalent attachment of biomolecules (e.g., proteins, amines, or amides) to improve bioselectivity [63]. Later in the study, we improved the design of the grafted layer by employing 3,4,5-tricarboxybenzenediazonium (ATA), which contains three carboxyl groups and promotes the formation of compact, high-quality monolayers. By systematically comparing mono- and tricarboxylated diazonium-functionalized surfaces, we investigated how grafting architecture and interfacial charge affects the electrochemical behavior of epinephrine (EP), a representative catecholamine neurotransmitter. Compared to previous studies that primarily focused on maximizing signal response or biomolecule immobilization using diazonium grafting, here, we demonstrate comparative molecular-based insights into the importance of chemical functionality and film morphology in the design of custom bio-sensitive interfaces.

## 2. Results and Discussion

**Figure 1:**
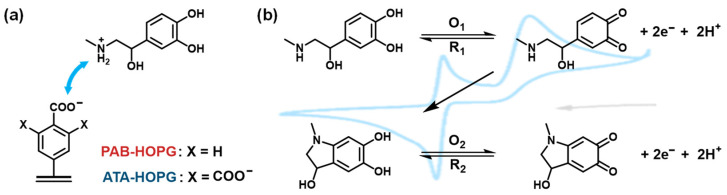
(**a**) Schematic illustration of the electrostatic recognition between protonated epinephrine (EP^+^) and surface carboxylate groups (COO^−^) on diazonium-functionalized HOPG electrodes at pH 7.4. PAB-modified surfaces (X = H) carry monocarboxylated aryl groups, while ATA-modified surfaces (X = COO^−^) feature tricarboxylated aryl groups that enhance ionic interactions with EP^+^. (**b**) Proposed two-step electrochemical oxidation mechanism of epinephrine in aqueous solution. The first redox couple (O_1_/R_1_) corresponds to the reversible oxidation of EP to epinephrinequinone. The second redox couple (O_2_/R_2_) involves intramolecular cyclization to form aminochrome-like species, which may undergo surface adsorption and further polymerization, especially on carboxyl-rich surfaces.

In this study, we investigated the electrochemical behavior of biomolecules on a precisely engineered substrate—a critical step towards the rational design of modern electrochemical biosensors. To minimize artifacts arising from the complex and poorly defined surface morphologies of practical sensors, we employed the basal plane of freshly cleaved HOPG, specifically the SPI-2 ZYB grade, characterized by its low-defect (Figure S1), atomically flat surface with high-quality graphene terraces extending up to 1 mm (mosaic spread: 0.8° ± 0.2°). To further tailor the surface, we covalently grafted ultrathin (<1 nm) monolayers bearing carboxylic acid (COOH) groups. These COOH functionalities (Figure 1a) are anticipated to interact with amine-containing bioanalytes via acid-base and hydrogen bonding interactions, thereby enhancing the specificity and sensitivity of the biosensor interface [46]. As a model bioanalyte, we selected EP, a representative catecholamine neurotransmitter. The electrochemistry of EP involves stepwise oxidation and chemical transformation processes (Figure 1b), which are often exploited for its detection. The electrochemical oxidation of EP proceeds via a two-electron, two-proton reaction to form epinephrinequinone (Figure 1b, O_1_/R_1_), followed by further intramolecular cyclization and polymerization steps that yield redox-active aminochrome-like and polyepinephrine species (O_2_/R_2_) [64]. Compared to freshly cleaved bare HOPG electrodes, carboxy-functionalized HOPG was hypothesized to exhibit enhanced sensitivity toward EP.

### 2.1. Electrochemical Grafting of PAB onto Graphite

**Figure 2:**
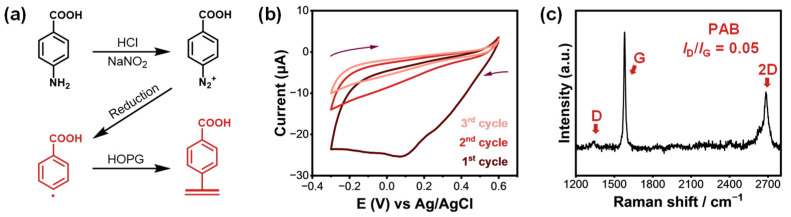
(**a**) Schematic illustration of the in-situ diazotization and electrochemical grafting of 4-aminobenzoic acid (PAB) onto HOPG, forming carboxyl-functionalized aryl layers. (**b**) Cyclic voltammogram of HOPG in a 2 mM PAB grafting solution over three successive scans, showing characteristic irreversible reduction peaks and a progressive decrease in current due to surface passivation. The arrows indicate the scan direction. (**c**) Raman spectrum of the PAB-grafted HOPG surface, displaying the D, G, and 2D bands. The low intensity ratio of D to G bands (I_D_/I_G_ = 0.05).

Building upon previous reports of enhanced sensitivity in carboxy-functionalized reduced graphene oxide (rGO) sensors compared to non-grafted controls [46], we developed our first model substrate by grafting para-aminobenzoic acid (PAB) onto HOPG via in situ diazotization [65]. An in situ diazotized 2 mM PAB aqueous solution was subjected to cyclic voltammetry (CV) to induce covalent modification on the HOPG surface. The electrochemical grafting was carried out in an in-house designed electrochemical cell with a fixed, well-defined exposure area to ensure reproducibility and reduce environmental interference. PAB was first converted to its aryl diazonium form in situ by adding sodium nitrite to its acidic solution, forming reactive aryl radicals upon electrochemical reduction (Figure 2a).

Figure 2b shows the representative CV curves during the grafting process. In the first cycle, a broad irreversible reduction peak was observed at +0.09 V vs. Ag/AgCl, corresponding to the reduction of the diazonium group and the formation of aryl radicals. The disappearance of this peak in subsequent cycles indicated the formation of a passivating non-conductive layer on the electrode surface, consistent with successful covalent attachment.

Raman spectroscopy, a sensitive technique for detecting structural defects in carbon materials, further confirmed the covalent functionalization. The pristine HOPG showed only characteristic G and 2D bands (1580 and 2800 cm^−1^, respectively (Figure S1)), while the PAB-modified HOPG exhibited an additional D band at 1336 cm^−1^, signifying the introduction of defects through sp^2^-to-sp^3^ hybridization (Figure 2c). The I_D_/I_G_ ratio of 0.05 for the PAB-grafted sample indicated a moderate degree of covalent modification. Raman results confirm the successful covalent functionalization of HOPG by PAB, denoted as PAB-HOPG.

### 2.2. Electrochemical Response of PAB-Modified Graphite to EP and AFM

**Figure 3:**
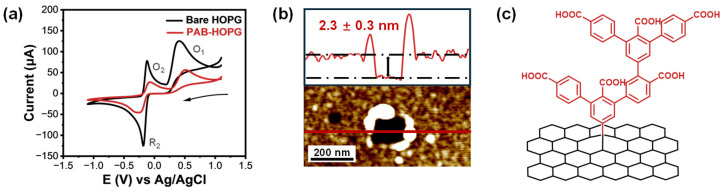
(**a**) Cyclic voltammograms of bare and PAB-grafted HOPG electrodes in 1 mM EP + 0.1 M PBS solution (pH 7.4). The PAB-modified surface exhibited a reduced redox current, indicating hindered electron transfer due to surface modification. The arrow indicates the scan direction. (**b**) AFM image and corresponding height profile of a scratch made on the PAB-grafted HOPG surface, revealing a multilayer aryl film with an average thickness of 2.3 ± 0.3 nm. (**c**) Schematic illustration of the multilayer growth mechanism during PAB grafting, where uncontrolled aryl radical coupling led to buried –COOH groups and partial passivation of the electrode surface.

After successful covalent modification with PAB, the sensing performance of the PAB-modified HOPG was evaluated using a custom-built electrochemical cell with a fixed exposure area to ensure measurement consistency. Both modified and unmodified HOPG electrodes were tested using the same substrate to eliminate batch-to-batch variation. CV tests were conducted in a 1 mM EP solution prepared in 0.1 M PBS (pH 7.4), pre-degassed with nitrogen for 2 h to remove dissolved oxygen. Surprisingly, as shown in Figure 3a, the redox current response of EP on PAB-HOPG is significantly lower than on bare HOPG, despite the introduction of carboxyl groups that could, in principle, enhance analyte affinity through specific electrostatic recognition interactions.

To elucidate the cause of the unexpected current reduction, nanoscale characterization was conducted using atomic force microscopy (AFM). Given the structural similarity between PAB and previously studied 4-nitrobenzenediazonium molecules [66] and previous reports of PAB grafting [65], we hypothesized the formation of multilayered organic films due to unblocked reactive sites on PAB.

AFM scratch tests revealed a layer thickness of approximately 2.3 nm on PAB-modified HOPG, indicating multilayer growth (Figure 3b,c), significantly exceeding the height of a single benzoic acid molecule. The overall film morphology, therefore, reflects an uncontrolled multilayered architecture, in which many –COOH groups are either buried or electrochemically inaccessible. This thick organic layer likely hinders electron transfer, thereby suppressing the EP oxidation current. The non-uniform and excessive film thickness was deemed detrimental for further sensing applications.

The performance loss in CV test is attributed to the dense multilayer film formed during PAB grafting, which obstructs charge transfer and sterically limits the interaction between EP and surface-bound –COOH groups. Together, these results indicate that while chemical functionality is present, its electrochemical utility is compromised by poor spatial accessibility. This finding highlights a key design trade-off: functional group density alone does not guarantee improved sensing performance if the film architecture inhibits interfacial charge transfer or molecular recognition.

### 2.3. Molecular Design and Electrochemical Grafting of ATA onto Graphite

**Figure 4:**
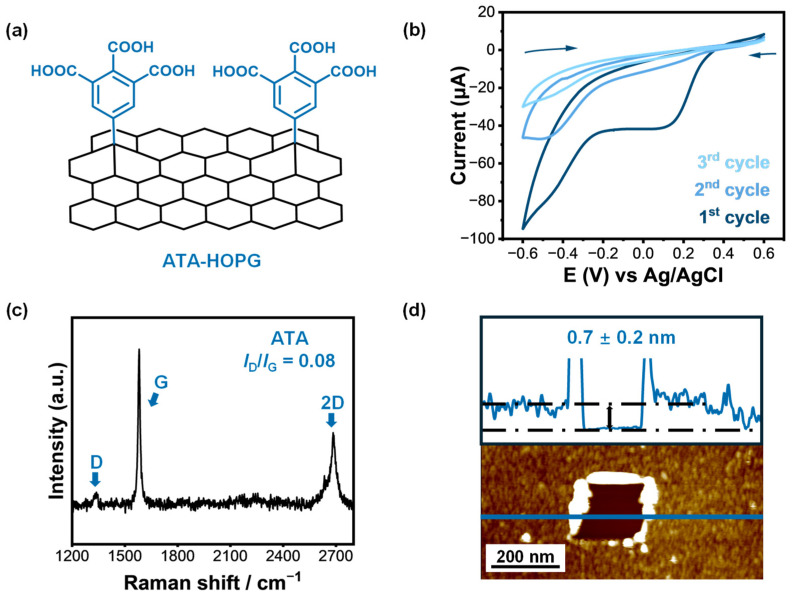
(**a**) Schematic representation of monolayer grafting of 3,4,5-tricarboxybenzenediazonium (ATA) onto the HOPG surface via electrochemical reduction. (**b**) Cyclic voltammograms of HOPG electrodes recorded in 2 mM ATA solution over three consecutive scans, showing a characteristic irreversible reduction peak and rapid current decay, indicative of self-limiting monolayer formation. The arrows indicate the scan direction. (**c**) Raman spectrum of ATA-grafted HOPG, exhibiting D, G, and 2D bands with a slightly increased D/G intensity ratio (I_D_/I_G_ = 0.08), suggesting minimal disruption of the sp^2^ lattice consistent with monolayer grafting. (**d**) AFM image and height profile of a scratch made on the ATA-modified HOPG surface, confirming the formation of a uniform organic film with an average thickness of 0.7 ± 0.2 nm.

Following the successful electrochemical grafting of PAB onto the HOPG surface under the established conditions, we sought to further control the grafting layer thickness by introducing bulky steric groups or protective substituents into the precursor molecules. For this purpose, we selected 3,4,5-tricarboxyphenyl diazonium salt (ATA) [67]. Compared to PAB, the ATA molecule bears three carboxylic acid groups on the aromatic ring at the 3-, 4-, and 5-positions. These substituents serve a dual function: (1) they sterically hinder multilayer growth, thereby confining the grafted organic layer to a uniform monolayer, and (2) they offer multiple functional sites for interactions with neurotransmitters such as EP (Figure 4a).

ATA-grafted HOPG samples were prepared using the same electrochemical protocol. In the CV profiles, a distinct reduction peak was observed at approximately +0.11 V vs. Ag/AgCl (Figure 4b), which corresponds to the one-electron reduction of the ATA diazonium moiety. The cyclic voltammetry of ATA grafting displays a distinct reduction peak in the first scan, followed by rapid current suppression in subsequent cycles. This self-limiting behavior is characteristic of surface-confined monolayer formation, where the initial aryl radicals passivate the electrode surface and inhibit further electron transfer.

Raman spectroscopy confirmed the successful surface modification. A pronounced D band emerged in the spectra of ATA-modified samples (Figure 4c), and the intensity ratio I_D_/I_G_ = 0.08 was slightly higher than that of the PAB-grafted counterpart. AFM scratch analysis (Figure 4d) revealed a uniform and compact organic layer with an average thickness of 0.7 ± 0.2 nm, consistent with a single molecular layer of tricarboxyphenyl groups grafted onto HOPG. While an increase in the D band intensity can result from both covalent functionalization and edge defects, the combination of AFM-determined film thinness and the electrochemical self-passivation behavior strongly supports the conclusion that this signal originates from well-dispersed monolayer grafting rather than multilayer or edge-induced disruption. These results confirm that ATA introduces a chemically active carboxylated interface while maintaining minimal thickness and high electrochemical accessibility—key features for preserving both recognition capability and charge transfer efficiency.

### 2.4. ATA-Modified Graphite Electrodes: CV of EP and pH-Dependent Surface Ionization

**Figure 5:**
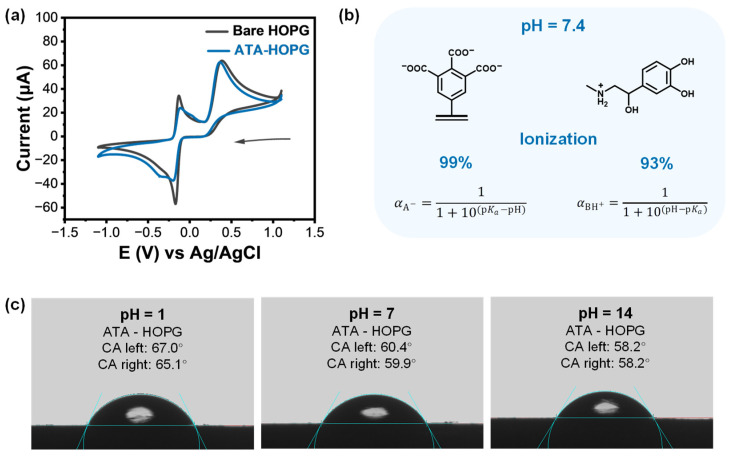
(**a**) Cyclic voltammograms of 1 mM EP recorded at bare and ATA-grafted HOPG electrodes in 0.1 M PBS (pH 7.4), showing comparable redox responses, indicating that the ATA monolayer retained electrochemical accessibility. The arrow indicates the scan direction. (**b**) Schematic illustration and calculated ionization states of EP and ATA at pH 7.4, based on the Henderson–Hasselbalch equation [68]. At physiological pH, EP was ~93% protonated (EP-H^+^), while the carboxyl groups of ATA were ~99% deprotonated (COO^−^), promoting strong electrostatic attraction. (**c**) Contact angle measurements of ATA-modified HOPG surfaces using aqueous droplets at pH 1, 7, and 14. A gradual decrease in contact angle from ~66° to ~58° was observed with increasing pH, consistent with enhanced surface hydrophilicity resulting from carboxylate deprotonation.

To evaluate the electrochemical performance of the ATA-modified interface, CV was performed in 1 mM EP solution (0.1 M PBS, pH = 7.4) using both bare and grafted HOPG electrodes. As shown in Figure 5a, the ATA-functionalized surface retained clear redox features of EP, including the oxidation peak at +0.37 V and a broad redox signal near +0.17 V (vs. Ag/AgCl), which corresponds to the reversible transformation of EP cyclization products. The peak intensities at both potentials remained comparable to those observed on bare HOPG, indicating that the thin organic layer does not hinder charge transfer.

To probe the interfacial ionic environment introduced by the carboxylated graft, we estimated the ionization states of both EP and ATA at physiological pH (7.4) using known pKa values (see Figure 5b and SI Sections S6 and S7 [69,70]). At this pH, EP exists predominantly as a cation (EP-H^+^, ~93% protonated), while the carboxylic acid groups of ATA are almost fully deprotonated (~99%, COO^−^), enabling strong Coulombic attraction at the interface.

To experimentally verify the presence of deprotonated carboxylates under neutral and basic conditions, we performed pH-dependent contact angle measurements. As shown in Figure 5c, the water contact angle on ATA-modified HOPG decreased with increasing pH—from ~66° at pH 1 to ~58° at pH 14—indicating enhanced surface hydrophilicity. This pH-responsive trend is consistent with the progressive deprotonation of carboxyl groups to carboxylate anions, which increase surface polarity and hydrogen bonding with water. For comparison, contact angle measurements were also performed on bare HOPG (Figure S4a,b). The HOPG surfaces showed negligible change in wettability across the pH range, confirming that the observed behavior on ATA-HOPG originates from the dynamic ionization of surface –COOH groups.

At low pH, the –COOH groups remained protonated, leading to lower molecular polarity and higher contact angles. As the pH increased, ionization yielded negatively charged –COO^−^ groups, enhancing hydrogen bonding with water and resulting in a lower contact angle. Notably, the most pronounced hydrophilization occurred at pH 7, consistent with the deprotonation of nearly all carboxylic groups at physiological conditions. These findings confirm that ATA grafts not only modify the surface chemistry of graphite but also impart dynamic, pH-responsive behavior that can be leveraged for biosensing applications. Compared to PAB, the sensitivity has improved but is still on par with HOPG. Using known pKa constants of EP and 1,2,3-benzentricarboxylic acid (as a close structural analog of ATA graft), we have estimated the ionization degree of EP and ATA at pH = 7.4. The calculations (Figure 5b and SI Sections S6 and S7) indicated that at this pH, the majority of carboxylic and amino groups are ionized. As an experimental verification that ATA grafts are indeed ionized, we have conducted pH-dependent contact angle measurements using bare HOPG and inert monolayer grafted TBD-HOPG as controls (Figure 5c and SI Section S4). It is clear that ATA grafts are effective in altering the surface properties of graphite, and that the largest hydrophilization already occurs when reaching pH = 7, which is understandable, since at this pH, almost all carboxylic groups have already been deprotonated into COO^−^ anions.

### 2.5. ATA-Modified Graphite Electrodes: DPV Sensing at Low EP Concentration

To further elucidate the sensing performance of the ATA-functionalized interface, differential pulse voltammetry (DPV) was employed due to its higher sensitivity and resolution compared to cyclic voltammetry—particularly useful for low-concentration analyte detection. DPV measurements were carried out over a decreasing series of EP concentrations, ranging from 1 mM down to 0.001 mM. ATA-modified HOPG and the corresponding bare HOPG surfaces were tested under identical conditions to enable direct comparison of interfacial behavior.

**Figure 6:**
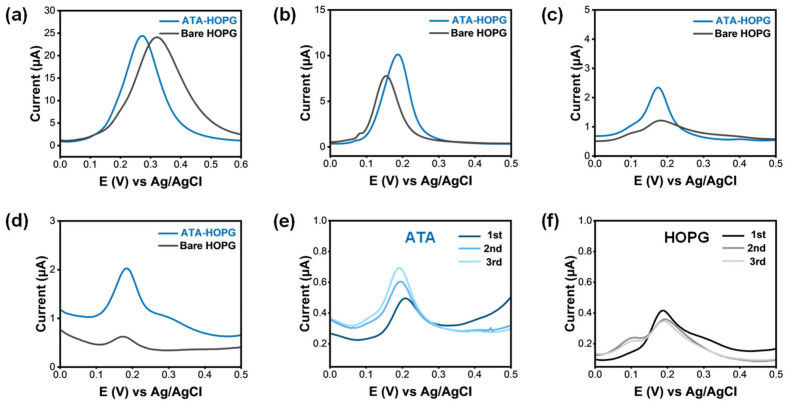
(**a**–**d**) Differential pulse voltammetry (DPV) responses of ATA-grafted and bare HOPG electrodes in 0.1 M PBS containing decreasing concentrations of EP: (**a**) 1 mM, (**b**) 0.1 mM, (**c**) 0.01 mM, and (**d**) 0.001 mM. ATA-HOPG exhibited consistently higher peak currents at lower EP concentrations, indicating enhanced sensitivity attributed to surface-enriched electrostatic recognition. (**e**,**f**) Consecutive DPV scans at 0.001 mM EP recorded on (**e**) ATA-grafted and (**f**) bare HOPG electrodes. ATA-HOPG showed a progressive increase in peak current over three scans, suggesting accumulation of EP or its redox products on the modified surface. In contrast, bare HOPG displayed minimal signal variation. These results underscored the role of molecular recognition and surface retention in signal amplification at the ATA-modified interface.

To minimize variability arising from substrate-to-substrate differences, each measurement was performed on a freshly prepared ATA-grafted HOPG sample. After recording the DPV response, the grafted layer was carefully removed using low-adhesion tape to reveal the underlying pristine HOPG, which was then re-tested in the same solution as an internal control. This approach ensured that performance differences could be attributed to the surface chemistry, rather than substrate quality. As shown in Figure 6a–d, both ATA-modified and bare HOPG electrodes exhibited decreasing peak current with reduced EP concentration, as expected under diffusion-limited conditions. However, a pronounced difference was observed at lower concentrations. At 0.01 mM, and especially at 0.001 mM EP, the ATA-functionalized surface delivered significantly higher oxidation currents than bare HOPG. This enhanced signal is attributed to surface-mediated preconcentration, arising from electrostatic attraction between protonated epinephrine (EP-H^+^) and the deprotonated carboxylate groups (COO^−^) on the ATA monolayer at physiological pH.

Notably, this enhancement was not evident at higher concentrations (e.g., 1 mM), where analyte diffusion dominates and surface interactions play a lesser role (see discussion in the next Section). The data thus confirm that interfacial ionic recognition becomes increasingly important under low-analyte conditions, enabling selective signal amplification. These findings demonstrate that ultrathin monolayer grafting not only preserves charge transfer efficiency but also enables ionic interaction-based enrichment, thereby enhancing electrochemical responses at biologically relevant concentrations.

### 2.6. Interfacial Recognition and Accumulation Processes on Carboxylated Graphite Electrodes

**Figure 7:**
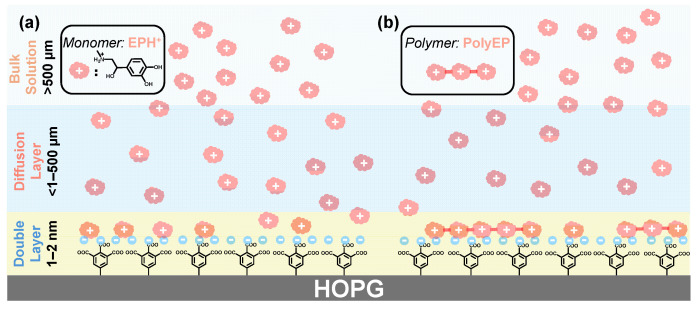
(**a**) At physiological pH (7.4), EP and the ATA-modified surface both existed in ionized forms—protonated EP (EP-H^+^) and deprotonated carboxylates (COO^−^), respectively—facilitating strong electrostatic interactions. This attraction promoted the local enrichment of EP near the electrode interface, enhancing recognition sensitivity at low concentrations. (**b**) During repetitive electrochemical cycling, redox-active EP species gradually accumulated on the electrode surface, forming polymeric layers stabilized through ionic anchoring to the ATA monolayer. This accumulation contributed to signal amplification upon repeated measurements. The monolayer structure of the ATA film preserved both molecular recognition capability and efficient electron transfer.

At physiological pH (7.4), EP exists predominantly in its protonated form (EP-H^+^), owing to the protonation of its primary amine group (pKa ≈ 8.6–9.0). Meanwhile, the carboxylic acid groups on the grafted aryl layers—particularly in the tricarboxylated ATA monolayer—are almost fully deprotonated under these conditions (pKa_1_–pKa_3_ ≈ 2.8–5.9), existing as carboxylate anions (COO^−^). This complementary charge pairing promotes electrostatic attraction between the electrode surface and the EP analyte, leading to interfacial enrichment at the sensor interface (Figure 7a).

At relatively high EP concentrations (e.g., 1 mM), diffusion from the bulk dominates the mass transport regime. In this case, even if surface-specific interactions exist, they are largely masked by the rapid replenishment of analyte at the electrode interface. Consequently, both bare and ATA-modified electrodes exhibit comparable voltammetric signals, as the contribution of interfacial preconcentration is relatively minor.

However, under dilute analyte conditions (e.g., ≤0.01 mM), the role of surface chemistry becomes more pronounced. As the driving force for analyte diffusion diminishes, localized interactions—such as ionic recognition between EP-H^+^ and surface-exposed COO^−^ groups—govern analyte accumulation and signal amplification. This is reflected in the enhanced DPV response of ATA-modified HOPG at low EP concentrations compared to the bare electrode (Figure 6a–d). To estimate the extent of interfacial enrichment, we compared the anodic peak currents of ATA-HOPG and bare HOPG under identical conditions. The current ratio (I_ATA-HOPG_/I_bare-HOPG_) consistently increased with a decrease in EP solution concentration (Table S1). This trend indicates that the role of electrostatic preconcentration becomes increasingly dominant at lower EP concentrations, where surface-specific interactions outweigh bulk diffusion effects. An estimated binding constant (≈1.6 × 10^6^ M^−1^, Section S8) of EP to ATA-HOPG approaches the binding affinities of variously designed carbon-based electrodes [71].

Moreover, when DPV is performed repetitively at 1 µM EP, a steady increase in current is observed for the ATA-functionalized surface (Figure 6e), in contrast to the relatively stable response seen on bare HOPG (Figure 6f). This progressive signal amplification can be attributed to the accumulation of EP oxidation products, which are known to undergo further polymerization to form redox-active polyepinephrine (polyEP) species [72,73]. These macromolecular products are insoluble and known to strongly adsorb on the electrode surface [68]. While their exact structure is unknown, from low concentration DPV experiments (Figure 6e,f) we hypothesized that these deep-oxidation products may carry additional positive charges or polar groups, promoting their retention at the interface (Figure 7b) of negatively charged ATA-HOPG electrodes. At higher EP concentrations (e.g., 1 mM), however, the poor solubility and adhesive nature of polyEP result in non-specific surface fouling on both bare [68] and grafted electrodes. This is confirmed by AFM measurements showing the formation of a ~3 nm polymer film on both surfaces following repetitive scans (Figure S6).

Together, these results suggest a two-step enrichment mechanism at ATA-modified electrodes: (1) Electrostatic preconcentration of EP-H^+^ monomers at low concentration through COO^−^/EP-H^+^ pairing. (2) Ionic retention of redox polymers through persistent Coulombic attraction and surface adhesion. This synergistic mechanism—enabled by the monolayer-thin, ionically active interface—demonstrates how surface chemistry and architecture can be tailored to enhance signal generation, particularly in sub-micromolar detection regimes relevant to bioanalytical applications [46,72,73].

While the absolute sensitivity of our system does not surpass that of nanomaterial- or enzyme-based EP sensors reported in the literature [74], it should be noted that the current platform is designed for the highly controlled fundamental study of structure-property relationships and rational design of bio-electrochemical interfaces. The monolayer-thin and chemically well-defined interface allows us to isolate the role of specific functional groups in governing recognition and accumulation. Insights obtained from such studies can lay a fundamental basis for the rational design of high-performance sensors.

Nevertheless, several potential limitations of the current system should also be acknowledged. First, under high EP concentrations or during repetitive measurements, the accumulation of poorly soluble oxidation products (above-mentioned “polyEP” species) can lead to surface fouling (see [68] and Figure S6). This fouling may give misleading measurements and deteriorate electrode performance over extended use. Second, while our study was conducted in controlled buffer solutions to isolate interfacial effects, the performance of the ATA-modified electrode in complex biological matrices (e.g., serum or cell lysates) remains to be explored. Matrix effects such as non-specific adsorption or ionic strength variation could impact recognition efficiency and signal reproducibility. Finally, although we observed stable responses over short-term measurements (e.g., multiple DPV scans at 1 μM EP), a more systematic evaluation of long-term stability and inter-day reproducibility would be necessary to assess the robustness of the system for real-world sensing applications. These aspects will be investigated in future studies as we aim to translate the current mechanistic insights into more application-oriented sensor platforms.

## 3. Materials and Methods

### 3.1. Materials

p-Aminobenzoic acid (C_7_H_7_NO_2_, ≥99%) and 3,4,5-tricarboxyaniline (C_9_H_7_NO_6_, ≥97%) were purchased from Macklin Biochemical Co., Ltd. (Shanghai, China). Sodium nitrite (NaNO_2_, ≥99%) was provided by China National Pharmaceutical Group Chemical Reagent Co., Ltd. (Shanghai, China). Hydrochloric acid (HCl, 36.0–38.0%) and acetonitrile (C_2_H_3_N, 99%) were obtained from Yonghua Chemical Co., Ltd. (Changshu, China). Sodium hydroxide (NaOH) and epinephrine hydrochloride (C_9_H_14_ClNO_3_, ≥98.0%) were purchased from Aladdin Biochemical Technology Co., Ltd. (Shanghai, China). Highly oriented pyrolytic graphite (HOPG, ZYB, 12 × 12 × 2 mm) was obtained from Bruker (Beijing) Scientific Technology Co., Ltd. (Berlin, Germany). A saturated Ag/AgCl reference electrode and platinum wire counter electrode were purchased from Tianjin Aida Hengsheng Technology Development Co., Ltd. (Tianjin, China). Atomic force microscopy (AFM) probes (Multi75Al-G) were provided by Oxford Instruments (Shanghai) Co., Ltd. (Oxford, UK). Low-adhesion tape (1 cm × 20 m) was purchased from Beijing Zhongjing Keyi Technology Co., Ltd. (Beijing, China). Phosphate-buffered saline (PBS, pH = 7.4, 0.1 M) dry powder was supplied by Shanghai Jizhi Biotech Co., Ltd. (Shanghai, China). Contact angle measurement syringes (1 mL, 0.25 mm inner diameter) were obtained from Shenzhen Zhijia Instrument Equipment Co., Ltd. (Shenzhen, China). Nitrogen gas (N_2_, 99.99%) was provided by Wujiang Guorong Gas Co., Ltd. (Wujiang, China).

### 3.2. Preparation and Cleaning of Covalently Grafted Samples on Graphite Surfaces

Fresh HOPG surfaces were cleaved using low-adhesion tape prior to each experiment. Electrochemical grafting was conducted in a custom-built single-chamber three-electrode cell, with HOPG as the working electrode, platinum wire as the counter electrode, and a saturated Ag/AgCl electrode as the reference. A 50 mM HCl aqueous solution was prepared using deionized water, in which the appropriate aniline derivative was dissolved at a concentration of 2 mM and ultrasonicated to ensure complete dissolution. An in situ diazotized 2 mM PAB aqueous solution was subjected to CV to induce covalent modification on the HOPG surface. The electrochemical grafting was carried out in an in-house designed electrochemical cell with a fixed, well-defined exposure area to ensure reproducibility and reduce environmental interference. The resulting solution was then mixed with a 0.1 M sodium nitrite solution at a 3:1 volume ratio and gently shaken. The mixture was introduced into the electrochemical cell as the electrolyte for the grafting reaction. After setting the cyclic voltammetry parameters, the electrochemical grafting process was initiated. Upon completion, the electrode was removed, soaked in NaOH solution, then transferred to hot acetonitrile (50 °C) for overnight soaking, followed by drying under nitrogen gas.

### 3.3. Material Characterization and Electrochemical Measurements

The surface morphology of HOPG was characterized using a Cypher ES atomic force microscope (AFM) equipped with Multi75Al-G probes operated in tapping mode. Data analysis was performed using the Asylum Research software (version 18.20.38, Oxford Instruments). All AFM images were subjected to second-order or higher polynomial flattening to eliminate background curvature.

To accurately determine the thickness of the grafted layer, the AFM scratch method was employed. A selected area of the image was first scanned in tapping mode, followed by switching to contact mode to mechanically remove the grafted molecules in the selected region without damaging the HOPG substrate. The system was then returned to tapping mode to rescan a larger area. The thickness of the grafted layer was determined by measuring the height difference between scratched and unscratched regions.

Raman spectroscopy was conducted using an HR800 laser confocal Raman spectrometer. A 633 nm laser was used with a 10× objective (NA = 0.90, working distance = 8 mm), laser power of 10 mW, and integration time of 5 s. A standard silicon wafer was used to calibrate the laser before each measurement to ensure accuracy.

The wettability of the surfaces was evaluated by measuring the contact angles of water droplets using an SL200 contact angle goniometer. Contact angles of different grafted surfaces were measured by dropping ultrapure water and calculating the angle formed. To study pH-dependent behavior, contact angles were recorded by dispensing droplets of water adjusted to various pH values. The HOPG surfaces were rinsed with ultrapure water and dried before each measurement.

Electrochemical tests related to biosensing were carried out using a CHI660E electrochemical workstation. HOPG served as the working electrode, Pt as the counter electrode, and a saturated Ag/AgCl electrode as the reference. A 0.1 M PBS solution was used as the supporting electrolyte. Epinephrine hydrochloride solutions of various concentrations were prepared and injected into the electrochemical cell. Prior to measurements, the electrolyte solution was purged with nitrogen to remove dissolved oxygen. Cyclic voltammograms were recorded by scanning the potential from −1.1 V to +1.1 V (vs. Ag/AgCl) at a scan rate of 100 mV·s^−1^. Differential pulse voltammograms were obtained by scanning from −0.10 V to +0.70 V with a pulse amplitude of 100 mV, a pulse width of 0.4 s, and a step potential of 1 mV. All electrochemical tests were performed at room temperature under ambient conditions.

## 4. Conclusions

In this study, we demonstrated the use of molecularly engineered electrochemical interfaces for clear-cut investigation and improvement of electrode–bioanalyte interactions. By grafting carboxy-functionalized monolayers, we demonstrated that tailored surface chemistry can amplify detection sensitivity, exemplified by the electrostatic and hydrogen-bond-mediated adsorption of EP. This approach not only advances EP sensing but also provides a blueprint for designing well-defined functionalized electrochemical interfaces targeting other biomolecules, emphasizing the broad potential of custom functionalization in biosensor development.

A critical insight from our findings is the necessity of nanoscale morphological control over grafted films. While dendritic structures (e.g., PAB) impair electrical responsiveness, sub-nanometer monolayers (e.g., ATA) optimized via molecular design ensure efficient charge transfer, highlighting the delicate balance between grafting density and structural precision required for high-performance electrodes. This principle extends beyond EP detection, advocating for rigorous material characterization in electrochemical systems where nanoscale architecture dictates functionality.

Furthermore, the interplay between detection methodology and operational parameters emerges as another key determinant of sensor efficacy. The superiority of DPV over CV in detecting trace EP underscores the need to align electrochemical techniques with analyte concentration and analyte–surface interactions. Such optimization, coupled with environmental controls (pH, temperature), reinforces the holistic approach needed to maximize sensor performance—a lesson applicable to diverse biosensing platforms.

Finally, the observation of EP-derived polymer accumulation on functionalized surfaces introduces an interesting preconcentration strategy, where multi-cyclic oxidation enriches analytes prior to quantification. This mechanism paves the way for ultrasensitive sensors leveraging iterative adsorption–oxidation cycles, transcending EP detection to enable trace analysis of neurotransmitters, environmental contaminants, or biomarkers. Collectively, this work bridges surface science, electrochemistry, and sensor engineering, offering foundational strategies for next-generation diagnostics and analytical technologies that demand both molecular specificity and nano/attomolar sensitivity.

## Data Availability

The original contributions presented in this study are included in the article/Supplementary Materials. Further inquiries can be directed to the corresponding authors.

## Supplementary Materials

The following supporting information can be downloaded at: https://www.mdpi.com/article/10.3390/molecules30102236/s1, Figure S1: Raman spectrum of pristine HOPG, Figure S2: Covalently Modified TBD-HOPG Samples, Figure S3: Cleaning Procedure for ATA-Grafted Samples, Figure S4: Control Experiment: Contact Angle, Figure S5: Electrochemical Current Changes Following CV Cycling, Figure S6: Surface Adsorption after Electrochemical Treatment. Table S1: Enrichment factors and corrected DPV peak currents on ATA-HOPG and bare HOPG at various EP concentrations.

## Abbreviations

The following abbreviations are used in this manuscript:

EP	Epinephrine
PAB	Grafts based on para-aminobenzoic acid
ATA	Grafts based on 3,4,5-tricarboxybenzenediazonium
HOPG	Highly oriented pyrolytic graphite
GO	Graphene oxide
CQDs	Carbon quantum dots
CNTs	Carbon nanotubes
rGO	Reduced graphene oxide
CV	Cyclic voltammetry
AFM	Atomic force microscopy
PBS	Phosphate-buffered saline
DPV	Differential pulse voltammetry
TBD	Grafts based on 3,5-bis-tert-butylbenzenediazonium
