# Supplementary material for: Towards a Rational Design of Biosensors: Engineering Covalently Grafted Interfacial Adlayers as a Testbed Platform for Electrochemical Detection of Epinephrine
Source: Molecules. 2025 May 21;30(10):2236. doi: 10.3390/molecules30102236 (PMC12114450; doi:10.3390/molecules30102236)
Supplement: Supplementary file 1 [file molecules-30-02236-s001.zip › molecules-3635566-supplementary.pdf]

Supplementary Material

# Towards a Rational Design of Biosensors: Engineering Covalently Grafted Interfacial Adlayers as a Testbed Platform for Electrochemical Detection of Epinephrine

**Xiaoli Chang [1], Yuan Fang [1,*] and Oleksandr Ivasenko [1,*]**

1 State Key Laboratory of Bioinspired Interfacial Materials Science, Institute of Functional Nano & Soft Mate-rials (FUNSOM), Soochow University, Suzhou 215123, China.

* Correspondence: yfang2000@suda.edu.cn (Y.F.); ivasenko@suda.edu.cn (O.I.)

**Table of Contents:**

**1. Raman spectrum of pristine HOPG**
**2. Preparation of Covalently Modified TBD-HOPG Samples**
**3. Cleaning Procedure for ATA-Grafted Samples**
**4. Control Experiment: Contact Angle Measurements of TBD-HOPG and Bare HOPG**
**5. Electrodeposition of EP oxidation products on the surface of electrodes**
**6. Deprotonation and Protonation Ratios at pH 7.4**
**7. Protonation Degree of Epinephrine at pH 7.4**
**8. DPV-Based Evaluation of Enrichment Efficiency****S1. Raman spectrum of pristine HOPG**

All the Highly Oriented Pyrolytic Graphite (HOPG, ZYB grade) used in the experiments was purchased from Bruker (Germany), with dimensions of 12 × 12 × 2 mm. For ZYB-grade HOPG with a mosaic spread of 0.8° ± 0.2°, the lateral grain size typically ranges from approximately 0.5 mm to 1 mm.

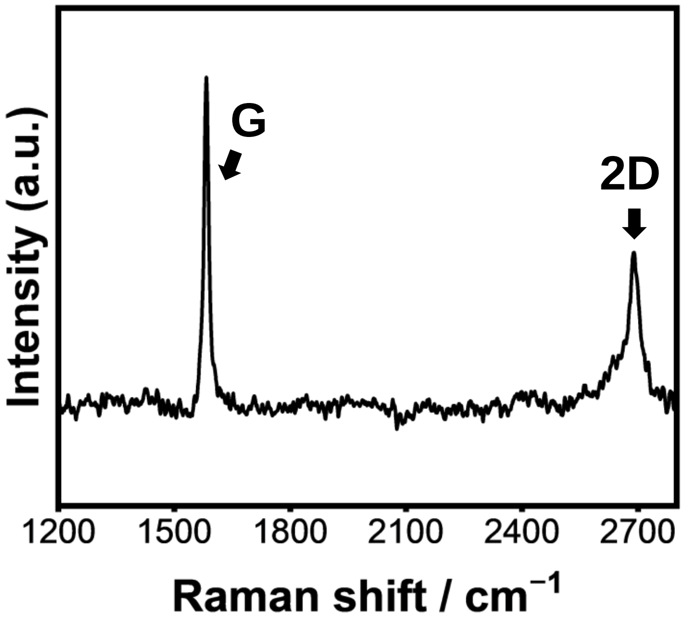


**Figure S1.** Raman spectrum of pristine HOPG.

**S2. Preparation of Covalently Modified TBD-HOPG Samples**

Electrochemical grafting of 3,5-bis-tert-butylbenzenediazonium (TBD) was performed using the same protocol as that applied to PAB and ATA. As shown in the CV curves in Figure S2(b), a similar electrochemical behavior was observed, indicating comparable grafting reactivity. A pronounced D band appeared in the Raman spectrum after grafting (Figure S2(c)), in contrast to the pristine HOPG surface, confirming the successful covalent attachment of TBD molecules. Due to the introduction of two bulky tert-butyl substituents, the grafted layer was limited to a monolayer, as revealed by AFM analysis in Figure S2(d).

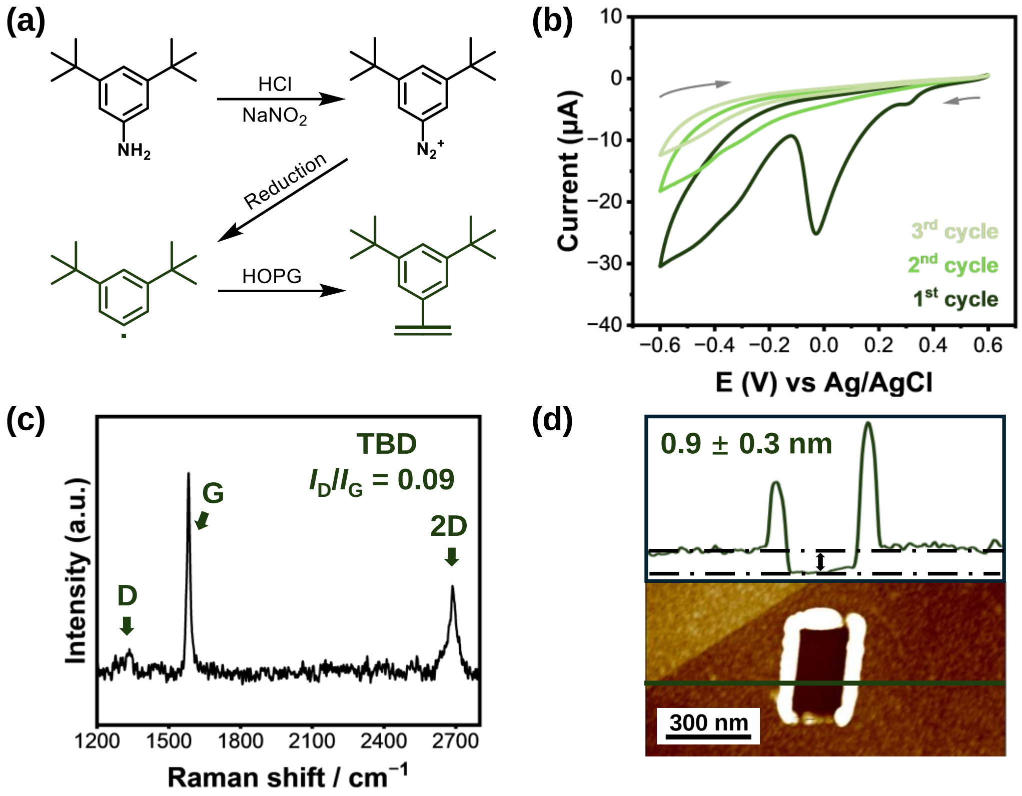


**Figure S2.** (a) Schematic illustration of the fabrication process for PAB-grafted HOPG. (b) Cyclic voltammogram of HOPG as the working electrode in a 2 mM TMP grafting solution, recorded at a scan rate of 30 mV $s^{-1}$. The arrows indicate the scan direction. (c) Raman spectrum of the TMP-grafted HOPG sample. (d) AFM image of the TBD-grafted HOPG surface.

## S3. Cleaning Procedure for ATA-Grafted Samples

In the case of the ATA-grafted samples, when the surface was rinsed only with acetonitrile after grafting, an apparent film thickness of approximately 1.8 nm was observed in the AFM image (Figure S3), indicating the presence of a physically adsorbed molecular layer. This excess layer was likely attributed to the deposition of byproducts, such as 1,2,3-benzenetricarboxylic acid. Due to the presence of carboxylic acid groups, intermolecular hydrogen bonding may have occurred between the grafted molecules and carboxyl-containing byproducts in solution, resulting in an increased apparent thickness. After soaking the sample in 1 M NaOH solution, the film height significantly decreased to approximately 0.7 nm, as shown in Figure 4(d).

Therefore, for both PAB- and ATA-grafted samples, the surfaces were first soaked in 1 M NaOH solution for 4 hours, followed by overnight immersion in acetonitrile at 50 °C to ensure complete removal of physisorbed species.

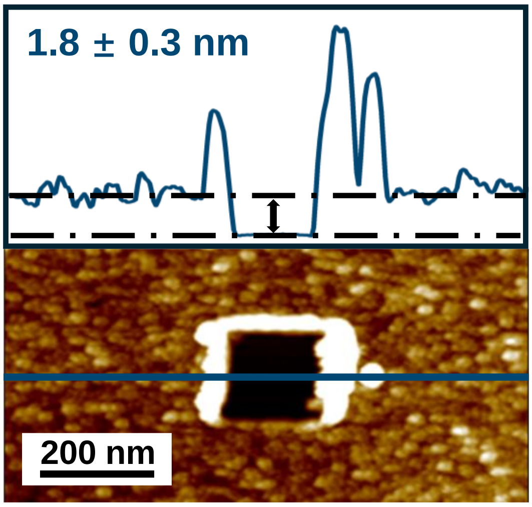


**Figure S3.** AFM image of the ATA-grafted HOPG sample after rinsing only with acetonitrile during the preparation process.

**S4. Control Experiment: Contact Angle Measurements of TBD-HOPG and Bare HOPG**

The surface of the ATA-grafted layer was rich in carboxylic acid groups (–COOH), whose ionization state varied significantly under different pH conditions, thereby influencing the surface hydrophilicity and charge distribution. In acidic environments, the carboxylic groups predominantly existed in their protonated form (–COOH), resulting in lower molecular polarity and a more hydrophobic surface, as reflected by a larger contact angle. This pH-dependent behavior was not observed for pristine HOPG or HOPG modified with TBD. For pristine HOPG, the contact angle gradually increased with increasing pH. In the TBD-modified samples, the contact angle was consistently higher than that of pristine HOPG at the same pH due to the strongly hydrophobic tert-butyl substituents on TBD molecules. However, the trend remained similar, with the contact angle increasing as pH increased. In contrast, ATA-modified HOPG exhibited an opposite trend. Owing to the presence of multiple carboxylic acid

substituents, surface deprotonation occurred under alkaline conditions, leading to a significant increase in hydrophilicity and a corresponding decrease in contact angle with increasing pH.

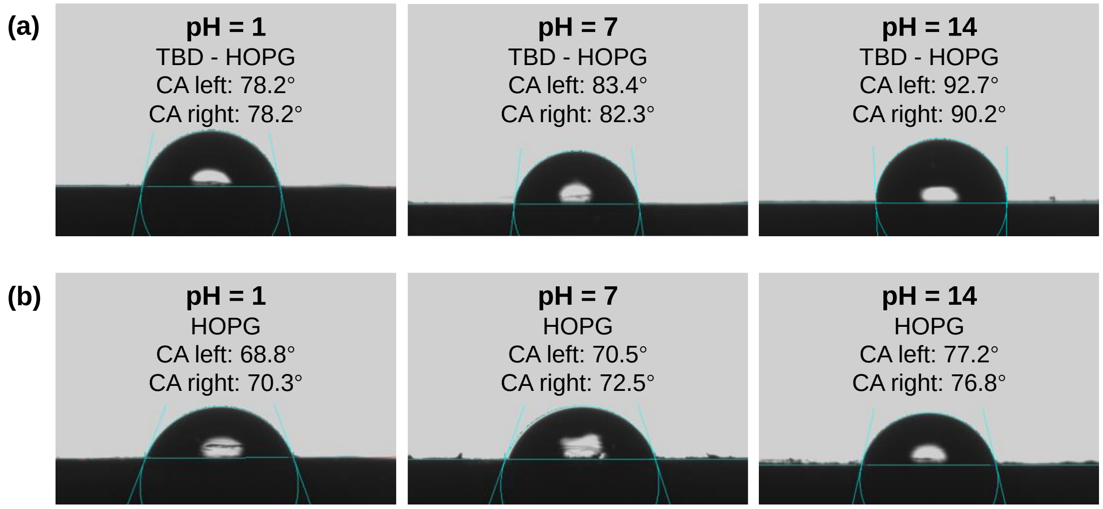


**Figure S4.** (a) Contact angle measurements of TBD-grafted HOPG using droplets with pH values of 1, 7, and 14. (b) Contact angle measurements of bare HOPG using droplets with pH values of 1, 7, and 14.

**S5. Electrodeposition of EP oxidation products on the surface of electrodes**

Continuous cyclic voltammetry (CV) scans in 1 mM EP + 0.1 M PBS aqueous solution were performed for both bare HOPG and ATA-modified HOPG electrodes, as shown in Figure S5. For both samples, the oxidation peak current corresponding to EP (denoted as O1) gradually decreased with increasing scan number, indicating progressive surface passivation. Peaks O2 and R2 were attributed to further redox processes of oxidized EP and its oxidized polymeric products respectively. With continued cycling, an increasing amount of electrochemical products accumulated on the electrode surface. Although this buildup contributed to the increase in the O2 peak current, it also hindered the oxidation of newly diffused EP molecules from the bulk solution, thereby reducing overall electrochemical efficiency.

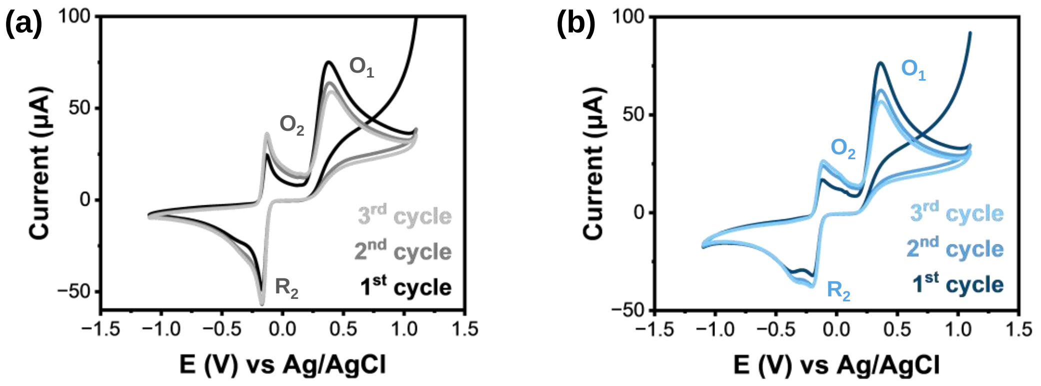

**Figure S5.** Cyclic voltammograms (CVs) of (a) bare HOPG and (b) ATA-grafted HOPG electrodes recorded in 1 mM EP +. Each electrode was scanned continuously for three consecutive cycles.

To further investigate the surface morphology after continuous scanning, both bare HOPG and ATA-modified HOPG were subjected to three successive CV scans in 1 mM EP solution. The electrodes were then removed, dried under a nitrogen stream, and analyzed using atomic force microscopy (AFM). As shown in Figure S6, a large number of physically deposited aggregates were observed on the surfaces of both samples, indicating significant accumulation of electrochemical products at this EP concentration.

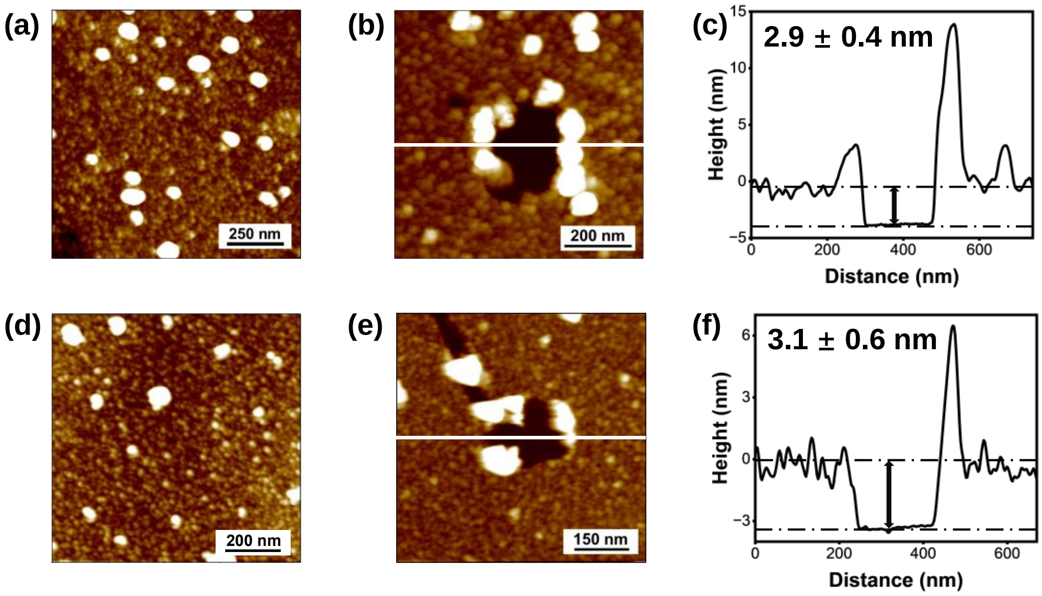


**Figure S6.** (a) Surface morphology of bare HOPG after three consecutive CV scans in 0.1 M PBS containing 1 mM EP. (b) Scratching test performed on the sample shown in (a) using AFM. (c) Height profile along the white line in (b). (d) Surface morphology of ATA-grafted HOPG after three consecutive CV scans under the same conditions. (e) AFM scratching test on the sample shown in (d). (f) Height profile along the white line in (e).

## S6. Deprotonation and Protonation Ratios at pH 7.4

Ionization and Protonation Ratios at pH 7.4:

Ionization Degree of 1,2,3-Benzenetricarboxylic Acid in PBS (pH 7.4)

1,2,3-Benzenetricarboxylic acid contains three carboxylic acid groups with the following pKa values [69].:

$pKa_1 = 2.80$

$pKa_2 = 4.20$

$pKa_3 = 5.87$

The ionization (deprotonation) degree refers to the average number of protons dissociated from the carboxylic acid groups at a given pH. The deprotonation fraction ($\alpha$) for each group was calculated using the Henderson–Hasselbalch equation:

$$\alpha = \frac{1}{1 + 10^{(pKa-pH)}}$$

At pH 7.4, the calculated deprotonation degrees are as follows:

For $pKa_1 = 2.80$:

$$\alpha_1 = \frac{1}{1 + 10^{2.80-7.4}} \approx 0.999975$$

For $pKa_2$ = 4.20:

$$\alpha_2 = \frac{1}{1 + 10^{4.20-7.4}} \approx 0.999369$$

For $pKa_3$ = 5.87:

$$\alpha_3 = \frac{1}{1 + 10^{5.87-7.4}} \approx 0.971333$$

The total ionization degree is the sum of the three values:

$$\text{Ionization degree} = \alpha_1 + \alpha_2 + \alpha_3 \approx 2.97$$

Thus, at pH 7.4, approximately 2.97 out of 3 carboxylic acid groups are deprotonated per molecule of 1,2,3-benzenetricarboxylic acid.

**S7. Protonation Degree of Epinephrine at pH 7.4**

The primary amine group in epinephrine has a reported pKa of 8.55[70]. The protonation fraction ($\alpha$) was calculated using the following form of the Henderson–Hasselbalch equation:

$$\alpha = \frac{1}{1 + 10^{\mathrm{pH}-\mathrm{p}K_a}}$$

For pKa = 8.55:

$$\alpha = \frac{1}{1 + 10^{7.4-8.55}} \approx 0.933886$$

Therefore, at pH 7.4, approximately 93.4% of the primary amine groups in epinephrine are protonated (i.e., exist in the $NH_3^+$ form).

**S8. DPV-Based Evaluation of Enrichment Efficiency**

It is possible to estimate the enrichment factor (EF) as the peak ratio of EP oxidation currents on ATA-grafted HOPG versus bare HOPG (EF = $I_{ATA\text{-}HOPG}/I_{bare\text{-}HOPG}$) under identical conditions (Table S1).

Table S1. Estimates of the enrichment factors together with background-corrected DPV peak currents measured on ATA-HOPG and on bare HOPG electrodes at different EP concentrations.

| EP Concentration (mM) | ATA–HOPG (μA) | Bare HOPG (μA) | EF = $I_{ATA\text{-}HOPG}/ I_{Bare\text{-}HOPG}$ |
|---|---|---|---|
| 1.0 | 23.54 | 22.77 | 1.03 |
| 0.1 | 9.81 | 7.29 | 1.35 |
| 0.01 | 1.72 | 0.68 | 2.53 |
| 0.001 | 1.12 | 0.31 | 3.60 |

This progressive increase in EF with decreasing analyte concentration illustrates the increase of relative contribution of adsorbed EP molecules compared to dissolved EPs available within the diffusion layer. Using 0.01mM and 0.001mM EP data we can estimate Kb, the binding constant of EP on ATA surface using modified Langmuir isotherm equation:

$$K_b = \frac{\theta}{C(1-\theta)} \approx \frac{I}{C(I_{max} - I)} \approx 1.6 \times 10^6 (M^{-1})$$

This binding constant is comparable to the $2.04\times10^6$ $M^{-1}$ affinity of EP towards carefully engineered bilayer lipid-like membrane supported on glassy carbon electrode [71].
